# Etiological factors in hallux valgus, a three-dimensional analysis of the first metatarsal

**DOI:** 10.1186/s13047-017-0226-1

**Published:** 2017-10-10

**Authors:** Tomohiko Ota, Takeo Nagura, Tetsuro Kokubo, Masateru Kitashiro, Naomichi Ogihara, Kenichiro Takeshima, Hiroyuki Seki, Yasunori Suda, Morio Matsumoto, Masaya Nakamura

**Affiliations:** 1 Departments of Orthopaedic Surgery, School of Medicine, Keio University, 35 Shinanomachi, Shinjuku-ku, Tokyo, 160-8582 Japan; 2 Departments of Clinical Biomechanics, School of Medicine, Keio University, 35 Shinanomachi, Shinjuku-ku, Tokyo, 160-8582 Japan; 3 Department of Orthopaedic Surgery, Tachikawa Hospital, Tokyo, Japan; 4 Department of Mechanical Engineering, Keio University, Yokohama, Japan; 5 Department of Orthopaedic Surgery, EIJU General Hospital, Tokyo, Japan; 6 Department of Orthopaedic Surgery, International University of Health and Welfare, Mita Hospital, Tokyo, Japan

**Keywords:** Hallux valgus, Foot, Metatarsal torsion, First metatarsal

## Abstract

**Background:**

It has been reported that hallux valgus (HV) is associated with axial rotation of the first metatarsal (1MT). However, the association between HV and torsion of the 1MT head with respect to the base has not been previously investigated. The present study examined whether there was a significant difference in 1MT torsion between HV and control groups.

**Methods:**

Three-dimensional (3D) computed tomography (CT) scans of 39 ft were obtained, and 3D surface models of the 1MT were generated to quantify the torsion of the head with respect to the base. The HV group consisted of 27 ft from 27 women (69.5 ± 7.5 years old). Only the feet of HV patients with an HV angle >20° on weight-bearing radiography were selected for analysis. The control group consisted of 12 ft from 12 women (67.7 ± 7.2 years old). In a virtual 3D space, two unit vectors, which describe the orientation of the 1MT head and base, were calculated. The angle formed by these two unit vectors representing 1MT torsion was compared between the control and hallux valgus groups.

**Results:**

The mean (± standard deviation) of the torsional angle of the 1MT was 17.6 (± 7.7)° and 4.7 (± 4.0)° in the HV and control groups, respectively, and the difference was significant (*p* < 0.01).

**Conclusions:**

This is the first study, to the best of our knowledge, to investigate 1MT torsion in HV patients using CT-based 3D analysis. The 1MT showed significant eversion in hallux valgus patients compared to control group patients.

## Background

Hallux valgus (HV) is one of the most common and significant diseases of the forefoot among elderly people [1–6]. In recent years, HV has been understood to be not only a deformity of the hallux, but also a deformity of the foot as a whole. The first ray forms the medial longitudinal arch, which absorbs the load, acts like a spring, and has a very important function during locomotion [7]. Therefore, deformities of the first ray readily disrupt the integrity of the foot structure, possibly leading to the onset of HV [8].

Many previous studies have been performed to investigate the pathogenesis of HV. It has been suggested that the shape of the head of the first metatarsal (1MT) is possibly related to the development of HV [9–14]. For example, Okuda et al. [13] demonstrated that there is a significant correlation between the roundness of the 1MT head (the so-called round sign) and HV using dorsoplantar radiographs. A more rounded lateral edge of the 1MT is possibly observed in HV because the lateral surface of the 1MT head can be viewed on a dorsoplantar radiograph if the entire 1MT is axially rotated in the everting direction. However, these previous studies only captured the shape of the 1MT head two-dimensionally using plain radiographs. No studies have compared the morphological differences of the 1MT and its head three-dimensionally between HV patients and a control group.

The apparent roundness of the lateral edge of the 1MT head on dorsoplantar radiographs could possibly occur because of axial torsion of the head of the 1MT with respect to the base of the 1MT. Torsion of the metatarsal head with respect to the base has been investigated previously in the field of physical anthropology [15, 16], but no studies have actually tried to identity the possible difference in 1MT torsion between HV and control groups. The present study, therefore, investigated the possible differences in torsion of the 1MT head between HV and control groups based on three-dimensional (3D) analysis using computed tomography (CT).

## Methods

### Patient selection

3DCT scan data of feet of HV patients and control subjects were obtained in the present study using a medical CT scanner (Aquilion 64, Toshiba Medical Systems Corporation, Otawara, Japan). Participants were outpatients attending the orthopedic clinic in Tachikawa Hospital (Tokyo, Japan). The HV group consisted of 27 ft from 27 Japanese women. The HV patients were diagnosed by one of the authors (T.K.) who is a foot and ankle orthopaedic surgeon having more than 21 years of experience. Only the feet of HV patients with an HV angle on weight-bearing radiography >20° were selected for analysis. The control group consisted of 12 ft from 12 Japanese women who had CT scans of the foot because of unilateral trauma or chronic foot disease, such as phalangeal, ankle, or calcaneus fractures, metatarsophalangeal joint bursitis, posterior ankle impingement syndrome, and second Lisfranc joint or ankle osteoarthritis. The same surgeon confirmed that the 12 healthy (unaffected) feet in the control group had no obvious foot and ankle pathologies, such as HV deformity, osteoarthritis, or sesamoid subluxation [17, 18]. The present study was approved by the Institutional Review Board of our institutions, and informed consent was obtained from all participants.

### Radiographic evaluation

**Fig. 1:**
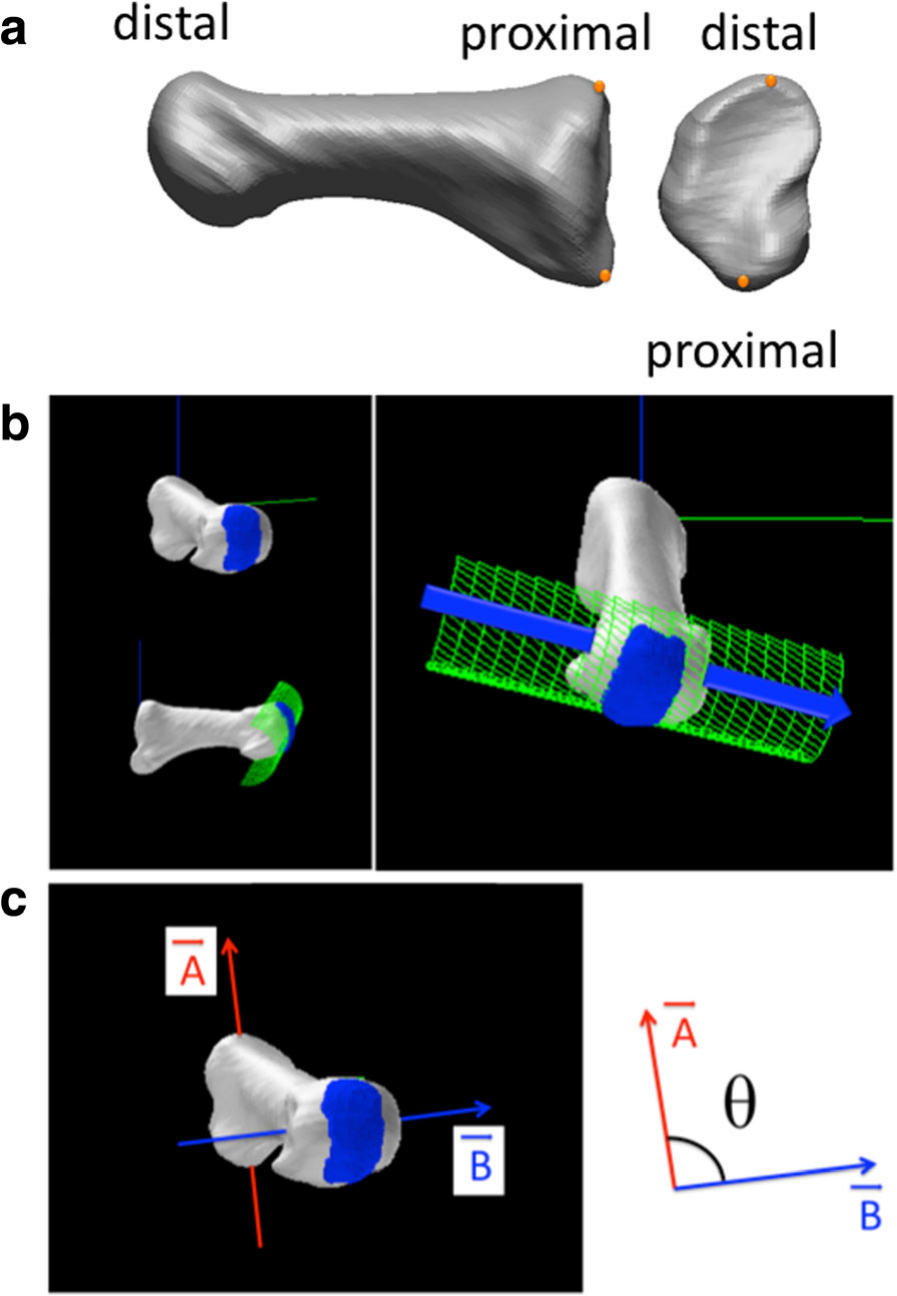
Quantification of the torsional angle of the first metatarsal head. **a** The surface model of the first metatarsal (1MT) is generated, and two characteristic points are chosen from the most dorsal and plantar edges of the proximal metatarsal surface to define the orientation of the base of the 1MT (vector A). **b** The articular surface of the metatarsal head is approximated by a cylindrical shape with a least-square method, and the orientation of the cylindrical axis in the direction of the fifth toe toward the hallux is calculated (vector B). **c** The angle between the vectors A and B (θ) minus 90° is defined as the torsional angle of the 1MT. The angle is positive for eversion

CT images were reconstructed at 0.5-mm intervals with a pixel size ≤0.682 mm. The 1MT was then reconstructed in a virtual 3D space using specialized software (Avizo®9.0; FEI Visualization Sciences Oregon, Hillsboro, OR, USA, and Rapidform®; 3D Systems, Inc., Rock Hill**,** SC, USA). Mirror image models were created for the left-side models so that all models could be treated as right-side models to facilitate comparisons. The most dorsal and plantar points on the edge of the proximal articular surface were digitized, and the dorsoplantar unit vector was calculated from these two points [15] (vector A) (Fig. 1-a). As for the distal articular surface, the median 1/3 of the articular surface of the 1MT head was extracted manually, and the surface was approximated by a cylindrical shape using a least-square method [19, 20]. The unit vector of the cylindrical axis in the direction of the fifth toe toward the hallux was calculated (vector B) (Fig. 1-b). The torsional angle of the 1MT was defined, following Drapeau et al. [15], as the angle between vector A and B minus 90°. The torsional angle of the 1MT is positive for eversion, i.e., the plantar portion of the articular surface of the 1MT head faces externally with respect to the base of the 1MT (Fig. 1-c). Only a proportion of the joint surface was used to quantify the orientation of the head in order to minimize the effects of possible deformities.

### Statistical analysis

The Shapiro-Wilk test of normality was used to evaluate the results. Differences in the torsional angle of the 1MT between the HV group and the control group were assessed by Welch’s two sample *t*-test. All statistical analyses were performed using SPSS statistics version 22.0 (IBM, Armonk, NY, USA).

### Evaluation of measurement

The inter- and intra-observer reliabilities for the torsional angle measurement were evaluated by having all CT data independently assessed by two of the authors who are foot and ankle orthopaedic surgeons (T.O. and M.K.) and by having one of the same authors (T.O.) re-assess the data after an interval of more than 1 year. The correlation coefficients were calculated to assess the inter- and intra-observer reliabilities using the same statistical software.

## Results

The intra- and inter-observer correlation coefficients for the present study were 0.975 (95% confidence interval, 0.953–0.987) and 0.966 (95% confidence interval, 0.935–0.982), respectively. The intra- and inter-observer errors (the mean value ± standard deviation) of the measurements of the torsional angle of the 1MT were 1.5 ± 1.1° and 1.9 ± 1.2°, respectively. These data indicated that the present measurement of the metatarsal torsion angles was highly reliable.

The mean (± standard deviation) age, body weight, and body mass index (BMI) of the HV group were 69.5 ± 7.5 years (range, 53 to 81 years), 55.2 ± 9.5 kg, and 23.7 ± 4.0 kg/m^2^, respectively. Those of the control group were 67.7 ± 7.2 years (range, 52 to 77 years), 58.0 ± 10.5 kg, and 24.6 ± 4.9 kg/m^2^, respectively. There were no significant differences in these mean values between the two groups (*p* = 0.49, *p* = 0.44, and *p* = 0.58, respectively). The mean (± standard deviation) HV angle in the HV group was 45.2 ± 9.3°. The corresponding values for the control group were not available, since dorsoplantar weight-bearing radiographs were not taken, since there was no clinical necessity for them in the control patients. Therefore, the non-weight-bearing HV angles of the feet were measured on the CT scans for comparisons. The values were significantly larger for the HV group (44.1 ± 8.5°) than for the control group (12.5 ± 3.7°). Sesamoid deviation was also evaluated based on the CT scans. Of the 27 HV feet, 18, 7, and 2 ft were classified as tibial sesamoid grades [18] of 3 (the entire tibial sesamoid is located lateral to the intersesamoid ridge), 2 (the tibial sesamoid is subluxated laterally but located under the intersesamoid ridge), and 1 (the entire tibial sesamoid is medial to the intersesamoid ridge), respectively. All 12 ft in the control group were classified as grade 1.

**Fig. 2:**
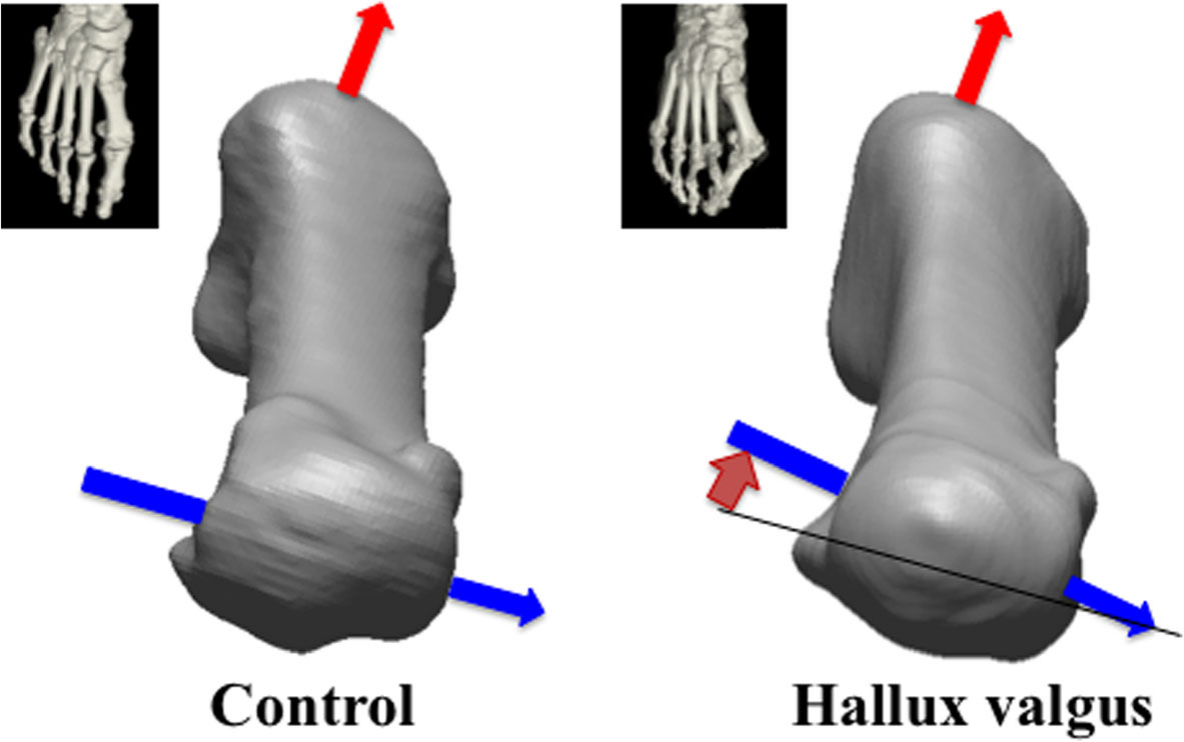
Comparisons of a typical 1MT between the control and HV groups. The torsional angle of the 1MT of the displayed 1MT is 8.6° and 36.2° for the control and HV groups, respectively

Figure 2 shows the 3D surface models of the two 1MTs representing the HV and control groups. As shown in Fig. 2, the 1MT head is more everted (pronated) with respect to the base in the HV group than in the control group. The torsion of the 1MT seemed to occur in the diaphyseal region, but not in the head region.

**Fig. 3:**
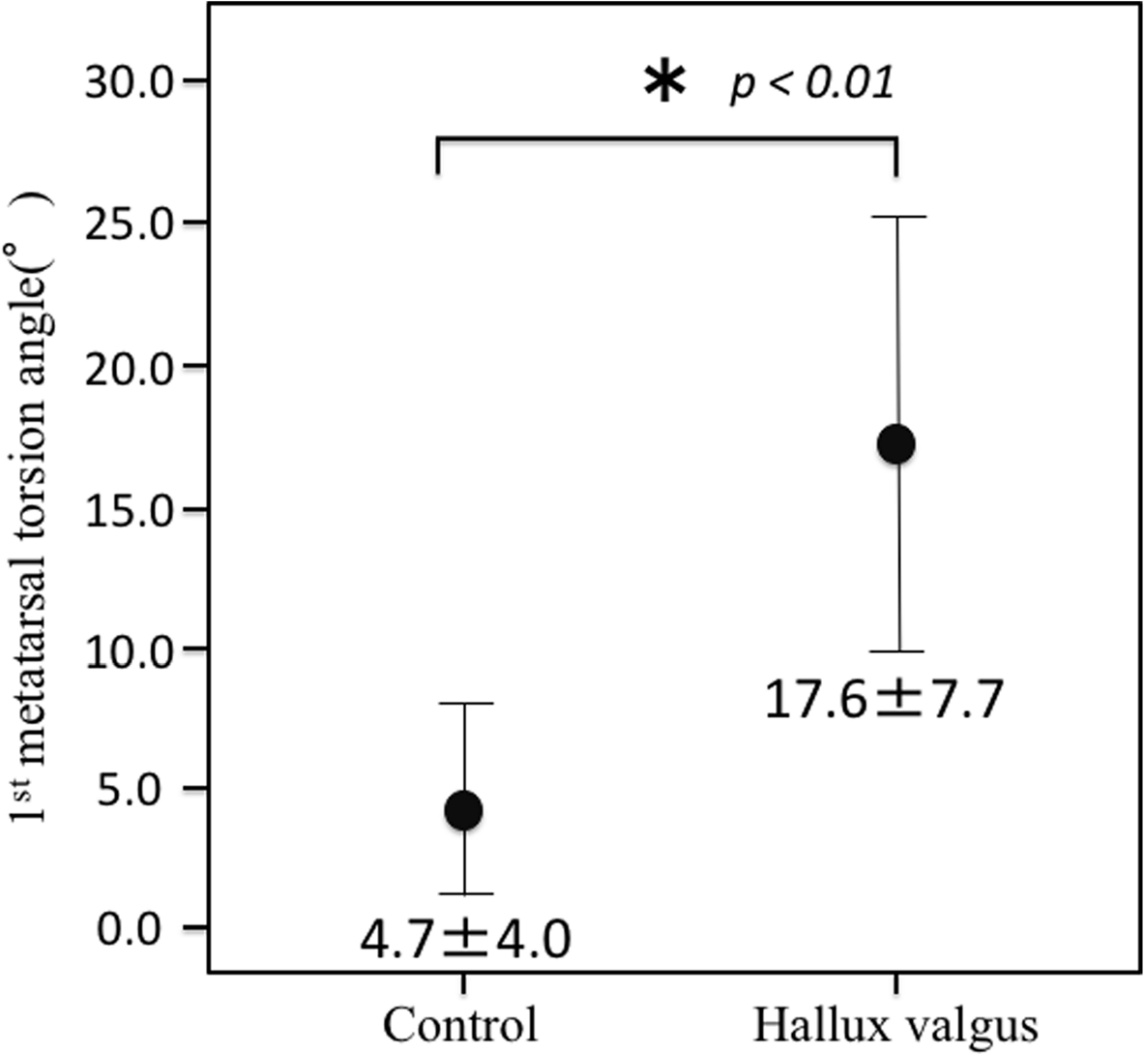
Comparison of the mean torsional angle of the 1MT between the control and HV groups. The mean torsional angle of the 1MT is significantly larger in the HV group (17.6 ± 7.7°) than in the control group (4.7 ± 4.0°; *p* < 0.01)

**Fig. 4:**
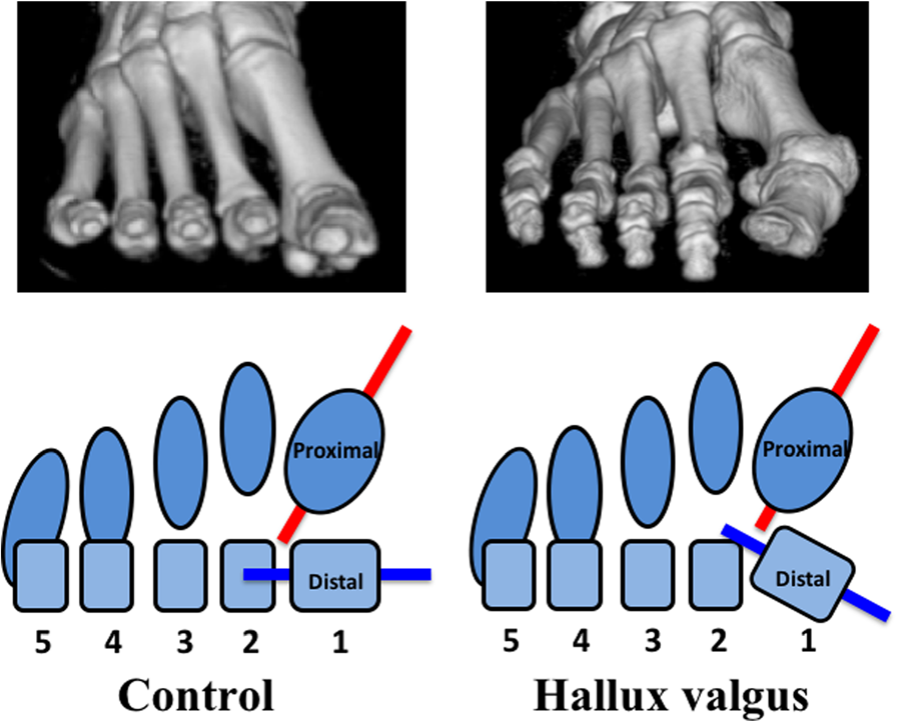
Schematic diagram of the present findings. The 1MT shows significantly greater eversion in HV patients than in control subjects. The articular surface of the head of the 1MT faces more externally with respect to the 1MT base in the HV patients than in the control subjects

The mean (± standard deviation) of the torsional angle of the 1MT were 17.6 (± 7.7)° and 4.7 (± 4.0)° in the HV and control groups, respectively (Fig. 3), and the difference was significant (*p* < 0.01). The 1MT head was significantly more everted in the HV group than in the control group (Fig. 4).

## Discussion

The results of the present study clearly demonstrated that the 1MT head showed significant eversion in the HV group compared to that in the control group. Previous studies using radiography have suggested that the 1MT is rotated in the pronated direction [21]. However, the present study clarified that the 1MT is not only rotated, but also twisted, in the HV group. The round sign is reportedly observed in HV patients because of larger axial rotation of the entire 1MT in the everting direction [13]. However, the present study suggested that the round sign can occur due to the torsional change in the morphology of the 1MT in HV patients. To the best of our knowledge, this is the first study providing quantitative evidence that the head of the 1MT is more everted with respect to the base in the foot of HV patients.

Since the present study only compared the torsion of the 1MT between the HV and control groups, it is difficult to assess possible mechanisms underlying the morphological change of the 1MT due to HV. However, such a change in morphology could possibly emerge both congenitally and developmentally. Some studies have suggested that HV occurs mainly due to genetic factors [22–24]. Therefore, the present result implies that the 1MT is congenitally more twisted in HV patients. If this is true, we could hopefully be able to predict the risk of HV by analyzing 1MT morphology. On the other hand, the change in the morphology of the 1MT might have occurred developmentally due to an age-associated decrease in force-generating capacity of the foot muscles and, hence, altered biomechanics of the foot. In particular, degeneration of peroneus longus could be a primary factor for the twisted 1MT, because the function of this muscle is to bring the proximal metatarsals into a closely packed position by everting the 1MT to stabilize the forefoot [25, 26]. Therefore, decreased pull of peroneus longus might alter the twisting moment generated in the 1MT, leading to adaptive change in the morphology of the 1MT. In fact, Kilmartin et al. [10] previously reported that there is no clear association between first metatarsophalangeal joint pathology including HV and the 1MT head shape in 10-year-old children. This finding, combined with the present finding, suggests that the change in the morphology of the 1MT might have occurred developmentally due to the altered biomechanics of the foot with aging. The causal relationship between the twisted 1MT and HV should be more rigorously investigated in future studies, but the present finding may contribute towards understanding of the pathogenesis of HV.

There are some limitations to the present study. First, the present study only compared the torsional angle of the 1MT of elderly people around the age of 70 years. The mean age of the two groups was not significantly different, and the feet used in the present analysis were all from female subjects, indicating that the observed difference is not due to age or sex differences. To help clarify the direct causal relationship between 1MT torsion and HV, age-associated changes in the torsion in the HV group should be investigated in future studies. Second, in the present study, the subjects were only adult women, because HV is much more frequent in adult women [1–6]. It has been reported that the mechanism for the occurrence of HV could be different for men and women [3]. Analyzing the differences in 1MT torsion in HV patients between male and female subjects may bring us closer to the pathology of HV. Third, the present study successfully quantified the torsion of the 1MT head with respect to the base of the 1MT, but it did not clarify where in the metatarsal the torsion occurs. Although it was visually observed that the torsion of the 1MT occurred in the diaphyseal region, but not in the head region, this must be quantitatively confirmed in future studies. Finally, the feet of the control group were actually not purely healthy in the present study, since it is difficult to collect age-matched subjects whose feet are healthy and with no foot disorders at that age. For the control group, subjects with any trauma or disorder in the first ray were excluded, and it was carefully confirmed that all 1MTs had no deformities or degenerative changes.

## Conclusions

This is the first study, to the best of our knowledge, to investigate 1MT torsion in HV patients using CT-based 3D analysis. The 1MT showed significant eversion in HV patients compared to control group patients.
